# Antibiotics Development and the Potentials of Marine-Derived Compounds to Stem the Tide of Multidrug-Resistant Pathogenic Bacteria, Fungi, and Protozoa

**DOI:** 10.3390/md18030145

**Published:** 2020-02-28

**Authors:** Justus Amuche Nweze, Florence N. Mbaoji, Gang Huang, Yanming Li, Liyan Yang, Yunkai Zhang, Shushi Huang, Lixia Pan, Dengfeng Yang

**Affiliations:** 1 Guangxi Key Laboratory of Marine Natural Products and Combinatorial Biosynthesis Chemistry, Guangxi Beibu Gulf Marine Research Center, Guangxi Academy of Sciences, Nanning 530007, China; justus.nweze@unn.edu.ng (J.A.N.); florence.mbaoji@unn.edu.ng (F.N.M.); hshushi@gxas.cn (S.H.); 2 Department of Science Laboratory Technology, Faculty of Physical Sciences, University of Nigeria, Nsukka PMB 410001, Nigeria; 3 Department of Pharmacology and Toxicology, Faculty of Pharmaceutical Sciences, University of Nigeria, Nsukka PMB 410001, Enugu State, Nigeria; 4 Guangxi Biomass Industrialization Engineering Institute, National Engineering Research Center of Non-food Biorefinery, State Key Laboratory of Non-Food Biomass, Guangxi Academy of Sciences, Nanning 530007, China; wangyi.07@163.com (G.H.); Lym810555@163.com (Y.L.); yangliyan.1988@163.com (L.Y.); 5 College of Life Science and Technology of Guangxi University, Nanning 530004, China; yykzhang@gxu.edu.cn

**Keywords:** marine, natural products, antimicrobials, drug-resistant, bacteria, fungi, algae invertebrates

## Abstract

As the search for new antibiotics continues, the resistance to known antimicrobial compounds continues to increase. Many researchers around the world, in response to antibiotics resistance, have continued to search for new antimicrobial compounds in different ecological niches such as the marine environment. Marine habitats are one of the known and promising sources for bioactive compounds with antimicrobial potentials against currently drug-resistant strains of pathogenic microorganisms. For more than a decade, numerous antimicrobial compounds have been discovered from marine environments, with many more antimicrobials still being discovered every year. So far, only very few compounds are in preclinical and clinical trials. Research in marine natural products has resulted in the isolation and identification of numerous diverse and novel chemical compounds with potency against even drug-resistant pathogens. Some of these compounds, which mainly came from marine bacteria and fungi, have been classified into alkaloids, lactones, phenols, quinones, tannins, terpenes, glycosides, halogenated, polyketides, xanthones, macrocycles, peptides, and fatty acids. All these are geared towards discovering and isolating unique compounds with therapeutic potential, especially against multidrug-resistant pathogenic microorganisms. In this review, we tried to summarize published articles from 2015 to 2019 on antimicrobial compounds isolated from marine sources, including some of their chemical structures and tests performed against drug-resistant pathogens.

## 1. Introduction

Antibiotics are a subcategory of that large group of chemical substances produced by microorganisms or made synthetically which include compounds that can inhibit the growth and even kill bacteria and other microorganisms in low concentration. Antimicrobials are the large group of chemical substances produced through natural (microorganisms, plants, and animals), semisynthetic or synthetic means and are effective against any microorganism (bacteria, fungi, virus, and parasite). These terms are used interchangeably today [1]. In 1929, the history of antibiotics began with the discovery of penicillin by Alexander Fleming and since then more antibiotics have been identified and some have been put to use. For more than six decades, antibiotics have remained unbeaten in their control of pathogenic disease-causing agents [2]. While some researchers continue to modify the existing antibiotics, more studies are focused on the discovery of new or novel antibiotics. The discovery and development of antibiotics with novel structural classes are very essential for some reasons. The resistance of microorganisms to available antimicrobials is on the increase, as is the toxic nature of some of currently available drugs, which limits their use. This is a serious threat to effective disease prevention and treatment of an ever-increasing range of infections caused by microorganisms including bacteria, parasites, viruses, and fungi [3,4].

Antimicrobial resistant pathogens are found in every country and we are fast running out of treatment options. This led WHO in 2017 to release a list of antibiotic-resistant "priority pathogens" that globally pose the greatest threat to human health (Table 1). The aim of publishing this list is to guide and promote research and development of novel antibiotics. The list is made up of a catalogue of 12 families of bacteria which WHO divided into three categories according to the urgency of the need for new antibiotics: critical, high, and medium priority (https://www.who.int/news-room/detail /27-02-2017-who-publishes-list-of-bacteria-for-which-new-antibiotics-are-urgently-needed). Other serious drug-resistant pathogens deliberately not included in the priority list are multidrug-resistant (MDR) or extensively drug-resistant (EDR) *Mycobacterium tuberculosis, P. falciparum* malaria, MDR *Candida* species (resistant to fluconazole, echinocandin, and amphotericin B), etc. This is because some of these pathogens such as *M. tuberculosis* are already a globally established priority for which innovative new treatments are also urgently needed [5,6].

One of the limitations of some effective antibiotics is their toxicity at or close to their therapeutic dose, thus providing another reason why the development of novel antibiotics with less or no adverse effects is of importance. For instance, amphotericin B is usually used to treat fungal infections caused by *Candida, Histoplasma* spp., *Cryptococcus*, and *Blastomyces*, but sadly it causes anorexia, nephrotoxicity, nausea, vomiting, and reduction of renal blood flow. Dermatophyte infections caused by *Microsporum, Epidermophyton*, and *Tricophyton* spp. are treated with griseofulvin antibiotic but its side effects are allergic reactions, headache, nausea, insomnia, and tiredness [7].

The mechanism of antimicrobial resistance, the reasons why antimicrobial resistance is on the rise, and different approaches and strategies employed in search of novel antimicrobials have been widely discussed in other reviews [8,9]. In this review, the authors voiced concern over the lack of advances in new antibiotics active against drug-resistant ESKAPE pathogens (*E. faecium*, *S. aureus*, *K. pneumoniae*, *A. baumannii*, *P. aeruginosa*, and *Enterobacter* species) and multidrug-resistant *Mycobacterium* species. As many researchers believe, the marine environment is an untapped potential source of antimicrobial agents, which could be effective against multidrug-resistant pathogens. A lot of progress has been made in this area for many years and there are many published articles on marine-derived antimicrobial compounds [3,8]. Some marine natural products (MNP) with antimicrobial potentials have been approved for use by US Food and Drug Administration as well as EU, and several other MNPs have entered phase 1, 2, and 3 clinical trials [3]. Only about 29% of the world surface is made up of land. The rest is ocean (nearly four kilometres deep on average), home to the marine habitats, which have different temperature and salinity, found in various locations, and is believed to contain organisms with much more extensive phylogenetic diversity. The association of these factors makes the marine habitats a unique and rich source of active compounds for drug production [10].

**Figure 1:**
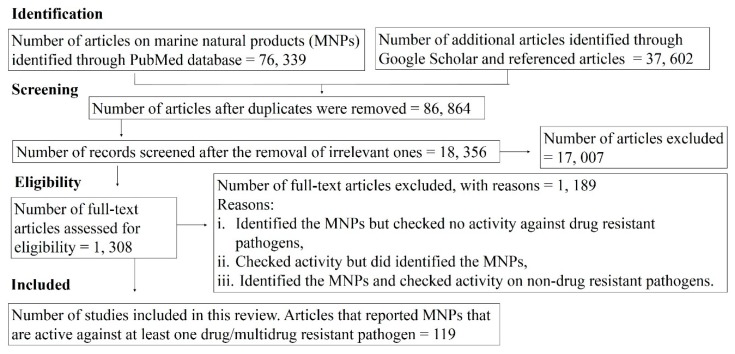
Flow chart of phases used to identify articles included in this review. Some of these articles were got using (marine (NOT military) OR marine natural products OR marine-derived) AND ((invertebrate OR sponge OR coral OR cnidarian OR arthropods OR echinoderms OR tunicates OR algae OR bryozoan)-associated (bacteria OR fungi OR algae))) ((drug-resistant OR multidrug-resistant) (bacteria OR fungi OR protozoa)) and careful insertion of (antibacterial OR antifungal OR antiprotozoal) and (oceans OR seas OR marshes OR bays OR shoreline OR estuaries OR deep sea OR coral reef OR coastal OR mangroves).

In this paper, we examined articles published (published in English language only) from 2015 to 2019 on marine natural products, structurally elucidated and tested against drug-resistant pathogenic bacteria, fungi, and protozoa (*Plasmodium* sp.). The articles used were found as shown in the flow chart in Figure 1 by searching PubMed/MEDLINE and Google Scholar using Boolean Operators (AND, OR, NOT) and a combination of related terms. Additionally, the search criteria were expanded using cross-referencing and “related articles” functions. About 119 articles were included in this review.

## 2. Marine Ecosystem as a Source of Antibiotics 

It is now obvious that the biological diversity of the ocean environment offers great promise as a source of antimicrobials for the future and could be potent against drug-resistant microbes, the “superbugs”. There are more than two hundred thousand described species of algae and invertebrates in the ocean, and representatives of every phylum (marine organisms are exclusively the twelve phyla) are found in the sea. Only a small percentage of the total number of species living in these habitats have been discovered and described [11,12]. The bacteria in the oceanic subsurface are believed to be up to ten percent of the total living biomass carbon in the biosphere. An appreciable number of chemical compounds from some of these species have been isolated but only a few of these compounds have been evaluated in clinically relevant bioassays. These diverse chemical compounds are used by the microorganisms mainly for defence and not basically for metabolic processes, and they confer some evolutionary advantage to them. These novel chemicals with pharmaceutical potential are being sourced from bacteria, fungi, invertebrate-associated microbes, algae, sponge, coral, cnidarian, arthropods, echinoderms, fishes, crabs, ascidians, molluscs, and bryozoan. Researchers are exploring different oceanic environments with different temperatures, tides, salt concentration, and depth [13,14]. 

Research in marine natural products has resulted in the isolation and identification of numerous diverse, as well as novel, chemical compounds with potent pharmaceutical importance. Some of these compounds have been classified into alkaloids, lactones, phenols, quinones, tannins, terpenes, glycosides, halogenated, polyketides, xanthones, macrocycles, peptides, and fatty acids [13,14]. All these are geared towards discovering and isolating unique compounds with therapeutic potential. 

## 3. Promising Marine-Derived Antimicrobial Agents against Drug-Resistant Microorganisms

### 3.1. Marine-Derived Antimicrobial Compounds from Bacteria

#### 3.1.1. Bacteria from Marine Sediments

An antibacterial compound, coumarin-6-ol, 3,4 dihydro-4, 4, 5, 7-tetramethyl- from *Streptomyces* VITAK1 exhibited activity against drug-resistant methicillin-resistant *Staphylococcus aureus* (MRSA) ATCC 33591 (IC_50_, 40 μg/mL) and other Gram-positive and Gram-negative bacterial pathogens [15]. A C-glycoside angucyclines-producing *Streptomyces* sp. SCSIO 11594 (South China Sea—depth of 2403 m) produced an additional two more new compounds. Aside from other biological activity, only dehydroxyaquayamycin (3) inhibited methicillin-resistant *Staphylococcus epidermidis* (MRSE) shhs-E1 (16.0 μg/mL), but was not active against other drug-resistant pathogens [16]. The derivatives of ikarugamycin (1–3) and four other known compounds produced by *S. zhaozhouensis* CA-185989 (Equatorial Guinea) showed activity against pathogens, including MRSA MB5393 (1–64 μg/mL) [17]. 

A strain of *S. rochei* PM49 (India) produced metabolite (sulfanyl cyslabdan-like compound) which had an inhibitory effect against MDR and ESBL-producing ATCC strains [18]. While another Indian bacterial isolate, *Streptomyces* sp. strain SMS_SU21 (7.5–30 cm depth), showed antifungal activity against *C. albicans* ATCC 10231 (0.5–5 mg/mL). GC-MS analysis of the crude extract of the isolated showed the presence of about sixteen compounds previously reported (Table S1) [19]. 

An antimalarial long-chain bicyclic phosphotriesters (Salinipostins A-K (1–11)) was produced by an obligate actinomycete, *Salinospora* sp. (US). Only salinipostins A (1) showed potent antimalarial activity by inhibiting chloroquine-resistant *P. falciparum* strain W2 at EC_50_ of 50 nM [20]. Polycyclic anthraquinones (N-acetyl-N-demethylmayamycin (1)) and a new phenazine ((-)-Streptophenazine B) were produced by *Streptomyces* sp. strain 182SMLY (East China Sea), which in addition to other potent activities, had an anti-MRSA effect against MRSA ATCC 43300 at minimum inhibitory concentration (MIC) of 20.0 μM [21] and 4.2 μg/mL [22], respectively. Interestingly, an angucycline antibiotics (Vineomycin A_1_ (1) and aquayamycin (3)) from *Streptomyces* sp. A6H inhibited the growth of MRSA ATCC 43300 (4.0 and 32 μg/mL), unlike its derivatives (2–9), and these compounds were not active against *C. albicans* ATCC 10231 (> 64 μg/mL) [23]. 

Anti-candida activities against *C. albicans* ATCC 10231 have been reported for metabolites produced by *Pseudonocardia endophytica* VUK-10 [24], *S. cheonanensis* VUK-A [25] (India), and *Micromonospora* sp. strain G019 [26] (Vietnam). These bacterial isolates respectively produced proline containing cyclic dipeptides (MIC, 8 and 64 μg/mL) (Table S1); 2-Methyl butyl propyl phthalate (1) (MIC, 8 ± 0.01 μg/mL) and diethyl phthalate (2) (MIC, 32 ± 0.02 μg/mL); and 1,4-dioxane derivative (2) and quinoline alkaloid (1) (MIC, 64 μg/mL). 

*Streptomyces* spp. (Southern Thailand) and *Micromonospora* sp. RJA4480 (85 m) (BarkleySound, British Columbia), produced compounds, fatty acids (Table 2) [27], and the first natural ansa macrolides [28] (Table 2), respectively, which at very low concentrations inhibited a similar pathogen, MRSA.

South China Sea sediment-derived *S. xinghaiensis* SCSIO S15077 (3536 m) produced antimicrobial compounds (new tunicamycin E (1) and other known compounds B, X, A, D_2_, C, and C_2_ (2–7)) potent against a fluconazole-resistant strain *C. albicans* ATCC 96901 (4.0–32 μg/mL) and other test pathogens [29]. *S. pratensis* NA-ZhouS1 (East China Sea, Zhoushan), subjected to metal stress, produced six angucycline polyketides, including stremycin A (1) and B (2), which inhibited MRSA (MIC, 16–32 μg/mL) and other pathogens [30]. This study has shown that heavy metal stress could effectively unlock biosynthetic genes in microbes responsible for production of novel antibiotics. Linear aminolipids (1, 2, 4, and 5) (bearing N-terminal glycine unit) produced by Antarctic *Aequorivita* sp, showed anthelmintic as well as antimicrobial activity against pathogens, including MRSA DSM 18827 (IC_50_, 22–145 μg/mL), unlike iso-pentadecenoic acid (3, 6, 7) that were inactive against MRSA (IC_50_, > 200 μg/mL) [31]. 

A Zhoushan Islands (Zhejiang, China) coastal soil-derived actinomycete, *Streptomyces* sp. ZZ446, in solid medium with sea salt, produced rare diketopiperazine glycosides (Maculosin and maculosin-O-a-L-rhamno- pyranoside (1, 2)), which inhibited MRSA (MIC, 37.0 μg/mL) [32]. However, previously, this bacterium in liquid medium with sea salt, produced streptopyrazinones A–D (1-4), N-acetyl-L-isoleucine-L-leucinamide (5), diketopiperazines (6–11), and alkaloid (12). Only the new compounds (1–5) were tested and they showed antimicrobial activity against pathogens, including MRSA ATCC 43300 at (MIC, 58.0–65.0 μg/mL) [33].

A promising therapeutic compound, di-(2-ethylhexyl) phthalate (DEHP) (phthalate derivative) produced by *B. subtilis* AD35 (Alexandria sea-shores, Egypt) showed antimicrobial activity against many pathogens including MRSA ATCC 43300 (25–32 mg/mL) but not *C. albicans* ATCC 10231 at the concentration used [34]. New bagremycin analogues F and G from coastal mud-derived bacterium, *Streptomyces* sp. ZZ745 had antimicrobial activity against MRSA ATCC 43300 (116.2–176.5 μM) [35]. 

More new antibacterial spirotetronates, phocoenamicins B (1) and C (2), including an already known compound phocoenamicin (3) synthesized by *Micromonospora* sp. CA-214671 (Gran Canaria, Spain) exhibited antimicrobial activity against some pathogens such as MRSA MB5393 and had poor activity against *E. faecium* (VRE) MB5570 [36]. An isolate from coastal area in Kappeln, Germany led to the isolation of dialkylresorcins from *Zobellia galactanivorans* OII3. These compound showed activity against strains of MRSA LT-1334 and MRSA COL (MIC, 4.0 μg/mL) [37]. Another German bacterium, *Labrenzia* sp. strain 011, produced two cyclopropane-containing medium-chain fatty acids (1, 2). Apart from inhibiting other bacteria, these compounds showed an inhibitory effect against strains of MDR MRSA LT-1338 and MRSA LT-1334 (2 and 3 mm) [38].

In 2018, marine-derived *Streptomyces* sp. XMA39 lead Jiang et al. to isolated new medermycin-type naphthoquinones strepoxepinmycins A–D and other known medermycin, which at low concentrations exhibited activity against fungi and bacteria, including MRSA ATCC 43300 (0.25–15 μg/mL) [39]. Di Zhang and his group isolated *Streptomyces* sp. ZZ741 (China) that produced new streptoglutarimides A−J (1−10) as well as a known streptovitacin A (11). Some of these compounds displayed antiproliferative properties, and antimicrobial activity against test pathogens which includes MRSA (MIC, 8−12 μg/mL) [40].

A Persian Gulf bacterium, *Bacillus velezensis* strain RP137 (70 cm), produced an aminoglycoside antibacterial substance (S-137-R) using low-cost rice starch and potassium nitrate as a fermentation medium. This new compound inhibited MRSA at MIC of 150 ± 5 μg/mL [12]. 

**Figure 2:**
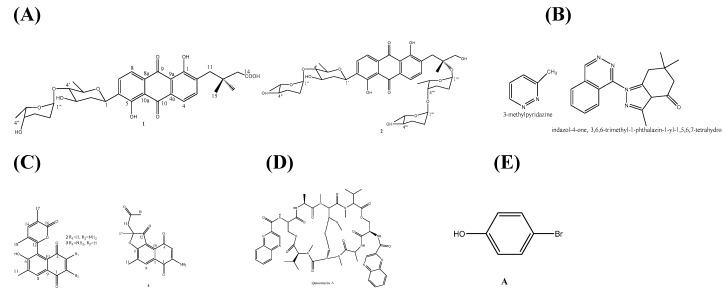
Structures of (**A**) Grincamycin L (1), and an angucycline derivative (2) [42]; (**B**) 3-methylpyridazine, indazol-4-one, 3,6,6-trimethyl-1-phthalazin-1-yl-1,5,6,7-tetrahydro- [43]; (**C**) Salinaphthoquinones B–D (2–4) [44]; (**D**) Echinomycin (quinomucin A) [45]; (**E**) 4-bromophenol [47].

The fermentation broth fraction of *Streptomyces* sp. SCA29 (India) had antibacterial activity against test bacterial pathogens, including MRSA ATCC NR-46171, and had other properties such as enzyme inhibitory and cytotoxic potentials. The spectral analyses led to the identification of an acetamide derivative, 4-methoxyacetanilide, as the bioactive compound [41]. An investigation by Yang et al. to discover novel antibiotics against multidrug-resistant pathogen led to the isolation of *S. lusitanus* OUCT16-27 from Indian Ocean (4495 m depth), which produced new angucycline, new grincamycin L (1), and other known compounds (Figure 2A). Only two compounds (1 and 2) exhibited moderate growth inhibitions against MDR pathogens tested (Table 3) [42]. Anti-MDR and anti-ESBL potential has been shown by the compounds produced by *Streptomyces* sp. Al-Dhabi-90 (Dammam, Saudi Arabia), which had an inhibitory effect against some pathogens and the chemical analysis of the broth revealed the presence of some compounds (Table 3) (Figure 2B) [43].

Recently, da Silva et al. isolated an obligate marine actinomycete, *Salinispora arenicola* (Brazilian Atlantic Ocean), and from the fermentation broth of the bacterium they identified salinaphthoquinones (Figure 2C), some of which had inhibitory activity against test drug-resistant pathogens (Table 3) [44].

A cyclic octapeptide-producing (quinoxaline) actinomycete, *Streptomyce* strain B475 (Guangxi Zhuang Autonomous Region, China) produced quinomycin A (Figure 2D) and other quinomycin derivatives that showed inhibitory activity against some drug resistant bacteria (Table 3). The mechanism of action of this antibiotic is to induce a DNA damage SOS response similar to levofloxacin [45]. An ansamycin-type polyketides-producing actinomycete, *Verrucosispora* sp. SCSIO 07399 (northern South China Sea at 2500 m depth) produced three new kendomycin analogues, kendomycins B (1), C (2), and D (3), which in addition to their moderately cytotoxic effects, exhibited good antimicrobial activity against pathogens, including MRSA shhs-A1 (MIC, 2.0, 1.0, and 4.0 μg/mL) [46].

A rare actinobacterial isolate, *Nocardiopsis* sp. strain SCA21 (Havelock Island, Andaman and Nicobar Islands, India), produced bioactive compounds (Figure 2E), which exhibited broad spectrum inhibitory activity against MRSA strains as shown in Table 3 [47]. 

#### 3.1.2. Bacteria from Marine Water Samples

Fatty acids produced by Red Sea bacterium, *Bacillus toyonensis* strain GAD1 (Alexandria, Egypt), exhibited antifungal activity against *C. albicans* ATCC 10231 (8–15 mm) [48]. Metabolites (hentriacontane, distearyl thiodipropionate and 1,2-benzenedicarboxylic acid, and mono-(2-ethylhexyl)-ester) produced by a similar halophilic bacterium, *Bacillus cereus* A30 (Rameswaram, India) exhibited activity on multidrug-resistant bacteria, MDR *E. coli*, MDR *K. pneumoniae*, MDR MRSA, and MDR *P. aeruginosa* (MIC = > 25, 25, 12.5, and < 25 μg/mL, respectively) [49].

#### 3.1.3. Marine Alga-Associated Bacteria

The extract of a red alga-associated bacterium, *Streptomyces* sp. MC025 (Kosrae, Micronesia), exhibited anti-biofilm formation by strains of *S. aureus*, including MRSA ATCC 33591. A series of bipyridines were isolated through bioactivity-guided method, among which collismycin C (2), was the most effective inhibitor (> 90%) of biofilm formation (50 µg/mL) [50]. Another bacterium, *Streptomyces* sp. HZP-2216E associated with a fresh sea green alga, *Ulva pertusa* (Shanwei City, Guangdong, China), which produced bioactive compounds which inhibited the growth of test pathogens, including MRSA ATCC 43300. Extensive chemical analysis led to the isolation of indolizinium alkaloid, streptopertusacin A, D, and bafilomycins D (MIC 40, 12.5, and 12.5 μg/mL) [51], and bafilomycin-type macrolides (MIC 7.4–33.1 μM) [52].

An actinobacterial strain identified as *Kocuria marina* CMG S2 associated with a brown macroalgae (*Pelvetia canaliculata*) (Karachi, Pakistan) was reported to produce a novel and potent antibiotic, kocumarin, which inhibited the growth of fungi and pathogenic bacteria, such as MDR bacteria strains of MRSA ATCC 33591, *S. pyogenes*, *S. typhi*, and *P. aeruginosa* at MIC of 10 μg/mL. This potency against MDR bacterial strains makes this antibiotic a good candidate for in vivo and other further studies [53]. Endophytic actinomycete, *Nocardiopsis* sp. GRG 2 (KT 235641), associated with macro algae (India), exhibited excellent anti-MDR and anti-ESBL activity pathogens, such as strains of MDR ESBL *P. aeruginosa* and *K. pneumonia* at MIC of 75 μg/mL. Based on chemical analysis, the fraction with anti-ESBL metabolites (Fraction 3) was confirmed to contain metabolite, named as 1, 4-diaza-2, 5-dioxo-3-isobutyl bicyclo[4.3.0]nonane (DDIBN) [54]. 

**Figure 3:**
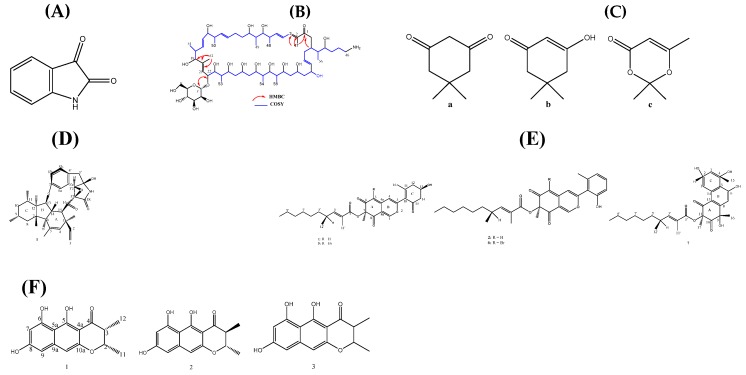
Structures of (**A**) desertomycin G (1) [55]; (**B**) isatin [69]; (**C**) TM1: 1, 3-dione-5,5-dimethylcyclo-hexane (a), 2-enone-3hydroxy -5,5-dimethylcylohex (b), and that of TM2: 4H-1,3-dioxin-4-one-2,3,6-trimethyl (c) [7]; (**D**) GKK1032C (1) [95]; (**E**) penicilones A and B (1, 2) (azaphilones), brominated azaphilones (5, 6), penijanthinones A (7) [96], and (**F**) monomeric naphtho-γ-pyrones, peninaphones A–C (1–3) [97].

Recently, a potent antibiotic-producing strain of actinomycete, *S. althioticus* MSM3 isolated from marine intertidal macroalgae (*Ulva* sp.) (Cantabrian Sea in Pedreña, Cantabria) led to the isolation of desertomycin G (Figure 3A), an antibiotic that possesses both antimicrobial and cytotoxic potentials. It inhibited the growth of MDR microorganisms in low concentration, as shown in Table 3. With these reported properties, desertomycin G will be a good candidate for further research, especially against drug-resistant pathogenic *M. tuberculosis* [55].

#### 3.1.4. Marine/Mangrove Plant-Associated Bacteria

*Streptomyces* sp JRG-04 (Tamil Nadu, India), which previously had potent antimicrobial activity at low MIC level concentration against various pathogens, including MRSA, produced benzoisochromanequinones polyketides that had a toxic effect on MRSA cell membrane and increased the number of dead MRSA cells [56]. These therapeutic properties make this strain a good candidate for various pharmaceutical applications.

#### 3.1.5. Invertebrate-Associated Bacteria (Sponge, Ascidian)

*Streptomyces* sp. LS298 (South China Sea) produced new cyclic dipeptide and echinomyci analogue which showed good inhibitory activity against drug-resistant pathogens (Table 2) [57]. An ascidian-associated *Streptomyces* sp. strain CA-271078 (SaoTome), produced in addition to other compounds, a new napyradiomycin (MDN-0170) (Table 2) which unlike others was not active against MRSA MB5393 at the highest concentration tested (> 64 μg/mL) [58]. In a co-culture experiment designed to induce production of antibacterial metabolites, *Streptomyces* sp. strain PTY087I2 associated with brown Panamanian tunicate (*Styela canopus*) from mangrove roots was co-cultured with other pathogens, especially MRSA, resulting strongly in enhanced antimicrobial activity against the test pathogens. The chemical analysis showed the presence of naphthoquinone derivatives, granaticin, granatomycin D, and dihydrogranaticin B [59]. 

The culture supernatant metabolite (protein CAP-1) of a jellyfish (Cyanea capillata) symbiotic bacterium, *Pseudomonas* sp. CMF-2, exhibited activity against *C. albicans* ATCC 10231 at MIC of 272 μg/mL [60]. A sea squirts-associated *Streptomyces* sp. ZZ338 (East China Sea) produced anti-MRSA compounds, actinomycins (1–3) (Table 2) [61]. *Streptomyces* sp. XY-FW47 obtained from a flatworm (*Paraplanocera* sp.) produced compounds that exhibited potent anti-MRSA ATCC 43300 activity and identified to be a series of geldanamycins (4,5-dihydro-17-O-demethyl geldanamycin (1), 2, 3, and 4) [62]. 

*S. albolongus* CA-186053, associated with an unidentified sponge (Bata, Equatorial Guinea), produced new medermycin derivative, named MDN-0171 (1), as well as another two known structurally similar compounds, medermycin (2) and antibiotic G15-F (3). The last two compounds inhibited the growth of MRSA MB5393 at a MIC value range of 2–4 μg/mL [63]. The marine sponge (*Callyspongia diffusa*) (southwest coast of India) had an associated *Bacillus tequilensis* MS145, which produced bioactive metabolite (pyrrolo[1,2-a] pyrazine-1,4-dione, hexahydro) active against MDR *S. aureus* (15 ± 0.172 mg/L) and other test pathogens [64]. Different researchers in 2018 independently isolated anti-MRSA bioactive compounds from two different deep-sea sponge-associated actinobacterium (WG1-60-61) [65] and sponge (*Theonella* sp.)-associated *Nocardiopsis* sp. HB-J378 [66]. Purified fermentation broths of these bacteria led to the identification of these compounds, urauchimycin D and nocardiopsistins A-C, respectively, which inhibited the growth of MRSA ATCC 700787 at MIC of 0.125 μg/mL and 3.12–12.5 μg/mL, respectively. 

Marine echinoderm (*Holothuria edulis*)-associated actinomycete, *Streptomyces* sp. G278 isolated from Cu Lao Cham-Quang Nam, Vietnam produced antimicrobial compounds (1–10) which inhibited a panel of clinically significant microorganisms. Compound 2 (3-hydroxyl-2-methylpyridine) and 5 (N-phenylnaphthalen-2-amine) exhibited antifungal activity against *C. albicans* ATCC10231 at MIC of 64 and 128 μg/mL, respectively [67]. New pyoluteorin analogues, mindapyrroles A−C(1–3) from shipworm-associated *P. aeruginosa* strain 1682U.R.0a isolated from gill homogenate of *Kuphus polythalamius* (Philippines) had antimicrobial activity against some test microorganisms, including MRSA ATCC 43300 at MIC value of 8, > 32, 4, 8, and 8 μg/mL, respectively [68]. 

Invertebrate-associated bacteria are very promising in the search for new antimicrobial compounds. The ethyl acetate crude extracts from liquid fermentation of tunicates–associated Gram-negative marine bacterium, *Pseudoalteromonas rubra* TKJD 22 (Indonesia) exhibited anti-MDR activity against some pathogens, including MDR *E. coli* and MDR-ESBL *E. coli*. The active compound in the fraction was identified to be a red-orange crystalline solid, isatin (Figure 3B) [69]. *Streptomyces* sp. G248 from marine Sponge (*Halichondria panicea*) (East Vietnam Sea) produced new antimicrobial lavandulylated flavonoids (1-3) and other known compounds (4–10). Only the new compounds (1–3) inhibited the growth of MDR *C. albicans* 10231 (Table 3) and other test microorganisms [70]. 

### 3.2. Marine-Derived Antimicrobial Compounds from Fungi

#### 3.2.1. Fungi from Marine Sediments

Wang et al. isolated two new benzoate derivatives, one new phenylacetate derivative and another known compound from *Engyodontium album* (at 2530 m in the Pacific Ocean). Only compound 3, ethyl 3,5-dimethoxy-2-propionylphenylacetate, had an inhibitory effect on MRSA ATCC 43300 at a MIC value of 7.8 μg/mL [71].

New compounds with anti-MRSA activity, according to Suga et al, named paraphaeosphaeride D (1) and berkleasmin F (2) together with known compound, berkleasmin A (3), were isolated from an artificial pond sediment fungus belonging to the Didymosphaeriaceae family, *Paraphaeosphaeria* sp. TR-022 (Machida city, Tokyo). This is in addition to their previously isolated fungal metabolites (biverlactones, aranorosin, and aogacillins). These compounds (1–3) not only enhanced anti-MRSA activity of Arbekacin (ABK) but also inhibited the growth of ABK-resistant MRSA TH-1466 and another 26 clinical ABK-resistant MRSA strains at MIC value range of 15.7–256 μg/mL [72].

The bioprospecting efforts of Alyssa L. Grunwald et al. led to the isolation of cyclic heptapeptide-producing fungal strain, *Mortierella* sp. RKAG 110 (Frobisher Bay, Nunavut, Canada). Sadly, these compounds, mortiamides A−D (1−4), did not exhibit any activity at the highest concentration used (> 128 μg/mL) against MRSA ATCC 33591 and VR *E. faecium* EF379 [73].

To show that standard laboratory cultures can hinder activation of specific gene clusters, which in turn hinder the production of metabolites with novel properties, Auckloo and his group’s addition of heavy metal cobalt (6 mM) to culture broth, in the form of “metal stress”, induced the production of new polyketides (bearing a migrated polyene chain) (Table 2). These three compounds derived from *Penicillium* sp. BB1122 (Zhoushan coast, China) inhibited the growth of MRSA at low concentrations [74]. Samples collected from Bohai Sea, China, led Xu et al. to isolate a diphenyl ethers-producing *Aspergillus ochraceus* LCJ11-102 sp. strain CUGB-F046. These compounds, diorcinol K (1), diorcinol D (2), diorcinol F (3), and diorcinol I (4), exhibited antibacterial activity against test pathogens including MRSA ATCC 700698 at MICs of 3.125, 6.25, > 50, and 6.25 μg/mL, respectively [75].

A study by Wang et al. isolated a deep-sea (4050 m) fungus, *Chaetomium* sp. strain NA-S01-R1 (West Pacific Ocean), that produced new chlorinated azaphilone polyketides (Table S1) which had antibacterial activity against strains of MRSA [76]. A new alkaloid-producing fungus, *A. sydowii* SP-1 from Antarctic marine sediment produced in addition to already known compounds (2–5), a new alkaloid, called acremolin C (1), which exhibited some inhibitory activity at low concentration (Table 2) [77]. A fungal strain, *Emericellopsis minima* strain A11 which produced an antibiotic called emerimicin IV was isolated from Talcahuano Bay (Chile). The fungal metabolite showed moderate activity against clinical isolates of MDR vancomycin-resistant strains of *E. faecalis* (MIC = 12.5 μg/mL) and MRSA (100 μg/mL) [78].

Six new diketopiperazines (1-3) and another known four compounds were isolated from the fermentation broth culture of the fungus, *A. versicolor* MF180151 (Bohai Sea, China). These compounds showed some levels of biological activity and only versicolorin B (5) and averufin (6) had inhibitory activity against MRSA [79].

Recently, a fungus, *Penicillium* sp. ArCSPf, from the eastern Arabian Sea ((Kochi transect, 500 m depth) produced a therapeutically active compound, (Z)-Octadec-9-enamide (oleamide), which exhibited an inhibitory effect against MRSA (MIC = 125 μg/mL) and other test pathogens. This fungus is a potential candidate which could produce more unknown bioactive compounds [80]. An aromatic polyketides-producing fungus, *Penicillium* sp. SCSIO 06720 (Indian Ocean), produced 5-[2-hydroxypropane-1-yl]-2,6-dimethlben- zene-1,3-diol (1) and coniochaetone L (2), together with another 19 known compounds (3–21). Only compound 16 exhibited moderate antibacterial activities, including against MRSA-shh-1 at MIC of 46.9 ± 29.7 μg/mL [81].

In addition to five known compounds produced by *Penicillium* sp. IMB17-046 (China mangrove swamp), new compounds (Figure S1) were identified, which had a broad-spectrum of antiviral and antibacterial activities. Only compounds 1 and 2 had inhibitory activity against drug-resistant *H. pylori* 159 at MIC values of 16 and 1 μg/mL, respectively [82]. Another anti-drug resistant metabolite (Figure 3C), inhibited MDR *H. pylori* as shown in Table 3. These compounds were produced by coastal sediment-derived *Trichoderma atroviride* strain KNUP001. Besides, the compounds showed some other interesting biological activities, which makes it a candidate for further in vivo animal model experiments [7].

A sea mud fungus, *Penicillium* sp. ZZ1283 (Karachi, Pakistan), synthesized 18 sesquiterpenes, including a new compound, named purpuride D (1) (analogue of drimane-type sesquiterpene lactones conjugated with *N*-acetyl-L-valine). These compounds (1–11, 15, and 18) except 12, 13, and 16, exhibited antimicrobial activity against pathogens, including MRSA at MIC values range of 4–14 μg/mL [83]. The extracts from rice fermentation medium of *Aspergillus* sp. strain SCSIO06786 led to the isolation and identification of fourteen compounds, which include new compounds such as a quinoline alkaloid (**1**), two bisabolane-type sesquiterpene derivatives (**2** and **3**), and a new natural product (**4),** along with another ten known compounds (**5**–**14**). Only compound 11–13 exhibited antibacterial activity against the test pathogens, including MRSA at MIC values of 3.13, 12.5, and 12.5 μg/mL [84]. 

#### 3.2.2. Fungi from Marine Water Samples

The ethyl acetate extract of fermentation broth of *A. sydowii* C1-S01-A7 (4950 m) from the West Pacific Ocean led to the isolation of two new xanthone derivatives (2-hydroxy-6-formyl-vertixanthone (1) and 12-O-acetyl- sydowinin A (2)) and another 12 known compounds. MRSA strains ATCC 43300 and CGMCC 1.12409 were inhibited by these two new compounds and other compounds (7, 8, 11, and 12) at a MIC value range of 15.4 ± 0.3–32.4 ± 0.5 μg/mL [85].

In 2017, Wang et al., in their search for bioactive metabolites, later isolated a citrinin-producing fungus, *Penicillium citrinum* NLG-S01-P1 (4650 m depth) that produced four citrinin dimer and nine monomer derivatives. The compounds exhibited biological activities and the two novel dimers (penicitol D (1) and 1-epi-citrinin H1 (2)) showed anti-MRSA activity against MRSA ATCC 43300 and MRSA CGMCC 1.12409 at IC_50_ value range of 7.3 ± 0.8–33.6 ± 0.2 μM. This study has added more compounds to the structural diversity of citrinin dimers [86].

#### 3.2.3. Marine Alga-Associated Fungi

The Mediterranean Sea green alga, *Flabellia petiolata,* sampled at Elba Island, harboured two fungal strains, including *Microascacea* sp. strain MUT 4861 and *Beauveria bassiana* strain MUT 4865. These researchers identified the components of the crude extracts of these fungal strains to be made up of several sphingosine bases, which may be responsible for their broad spectrum of antibacterial activity against MDR pathogens (Table S1) [6]. 

#### 3.2.4. Marine Plant-Associated Fungi

*P. sclerotiorum* M-22 from rotted leaf (Hainan province, China) synthesized azaphilone compounds, penicilazaphilone B and C. These compounds exhibited potent biological activities, including the inhibition of ESBL *K. pneumoniae* ATCC 700603 by Compound B and C at MIC values of 500 and 15.63 μg/mL, respectively [87]. An endophytic fungus of *Nymphaea nouchali* (Sri Lanka) called *Chaetomium globosum,* led to the isolation of cytochalasans, chaetoglobosin A and C from the crude extract of the two distinct fungi. The compounds showed antibacterial activities against test microorganisms, and chaetoglobosin A (1) inhibited MRSA ATCC 33591 and *S. aureus* ATCC 43300 at MIC values of 32 and 32 μg/mL, respectively [88].

A fungus, *Pestalotia* sp., associated with the mangrove plant, *Heritiera fomes* (Bangladesh), exhibited antimicrobial activities against pathogens, including strains of MRSA (32–128 μg/ml). Examination of the crude extract fractions revealed the presence of xylitol (1) in the ethyl acetate extract and oxysporone (2) in methanol extract [89].

The rhizosphere has been a dynamic region governed by complex interactions between microorganisms and plants. The pattern and composition of root exudates affected microbial activity and population numbers. Chen et al. isolated mangrove rhizosphere soil-derived fungus, *Penicillium janthinellum* HK1-6 (Hainan Island, China), that produced new azaphilones, penicilones A−D (1−4), from which other compounds (parental azaphilones, E (5) and F (6)) were derived through ester hydrolysis of compounds 2 and 4. As an anti-MRSA, MRSA ATCC 43300, MRSA ATCC 33591, and VR *E. faecalis* ATCC 51299 were inhibited by compounds 2-4 at a MIC value range of 3.13–6.25 μg/mL [90].

A South China Sea mangrove *Brguiera sexangula* var. *rhynchopetala*-derived fungus, *Daldinia eschscholtzii* HJ001, produced a new cytochalasin metabolite and another three known compounds (2–4). Only the compound, 1, [11]-cytochalasa-5(6),13-diene-1,21-dione-7,18-dihydroxy-16,18-dimethyl- 10-phenyl-(7S*,13E,16S*,18R*), exhibited weak antibacterial activity against MRSA ATCC 33591 at a MIC value of 50 μg/mL [91]. In addition to their previous findings, plant (*Bruguiera gymnorrhiza*) endophytic fungus, *Penicillium* sp. GD6 (Zhanjiang, China) synthesized two new compounds, sorbicillin derivative (1) and diketopiperazine alkaloid (2), in addition to five others (3−7) already identified. Only one compound (1) had inhibitory activity against MDR MRSA (MIC = 80 μg/mL) [92]. 

An endophytic *Cladosporium* sp. JJM22 (South China Sea) synthesized new ribofuranose phenol derivative, including six other known compounds (3–8). Of all the compounds tested for antimicrobial activity, only compound 3 ((3S)-3,8-dihydroxy-6,7-dimethyl-α-tetralone (3)) showed broad inhibitory activity, including against MRSA CMCC(B) 63303 (20 μM) [93]. Similarly, a sesquiterpene-producing Chinese plant endophytic fungus, *Cytospora* sp., prodcued a new biscyclic sesquiterpene seiricardine D (1), and another eight known metabolites. Some of these compounds exhibited antimicrobial activities against other pathogens but only compound 5 ((22E, 24R)5, 8-epidioxy-5a, 8a-ergosta-6,22E-dien-3ß-ol (5)) showed a moderate inhibitory effect against MRSA GIM1.771 (233.3 μM) [94].

*Penicillium* sp. CPCC 400817 yielded one more new alkaloid (GKK1032C (1)) (Figure 3D) and another four known alkaloids (pyrrospirones E (2), F (3) [7], GKK1032B (4) [8], and A2 (5)). Aside from GKK1032C (1), which was potent against MDR MRSA at a low concentration, other compounds had no inhibitory activity against Gram-negative bacteria such as ESBL *Escherichia coli* 46, and carbapenem-resistant bacteria [95]. An addition of NaBr instead of sea salt to the fermentation culture of this fungus led to the isolation of brominated azaphilones, new tricyclic polyketides, and two known penicilones (Figure 3E). Compared to the chloro analogues (3, 4) previously produced, these new brominated azaphilones (5, 6) had the opposite configuration at C-7. Compound 5, 6 and 7 exhibited antimicrobial activity against MRSA ATCC 43300 and ATCC 33591, and VR *E. faecalis* ATCC 51299 (3.13–25 μg/mL) [96]. 

Another related fungus, *Penicillium* sp. HK1-22, isolated from the same location produced 3 monomeric naphtho-γ-pyrones (Table 3) (Figure 3F) and another 2 known bis-naphtho-γ-pyrones (4 and 5). However, compounds A–C had no activity against VR. *E. faecium*, but showed moderate inhibitory activity against MRSA strains and other pathogens [97].

*Taeniolella* sp. BCC31839 isolated from a wood (family Poaceae) in a mangrove forest, Bangkok, Thailand, synthesized two unknown enantiomeric chromone derivatives, and another 6 known compounds. These two novel compounds (R)- and (S)-taeniolin (1 and 2) exhibited no inhibitory activity against MDR *P. falciparum* but lateropyrone (3) inhibited the same malaria parasite at a MIC value of 9.75 μ/mL [98].

#### 3.2.5. Invertebrate-Associated Fungi (Sponges, Ascidians, Crab)

Antibacterial compounds talaromycesone A and B (oxaphenalenone dimers), produced by a sponge (*Axinella verrucosa*)-associated fungus, *Talaromyces* sp. strain LF458, was collected at the Mediterranean Sea (Italy). The compounds were active against pathogens, including MRSA at IC_50_ of 4.6 μM [99]. Two marine fungal strains (LF327 and KF970) in the family Lindgomycetaceae that produced an unusual polyketide were isolated from a sponge (*Halichondria panicea*) of the Baltic Sea (Kiel Fjord) and the Antarctic. The new polyketides, lindgomycin (1) and ascosetin (2), possess an unusual carbon skeleton, such that the bicyclic hydrocarbon and a tetramic acid (two distinct domains) are joined by a bridging carbonyl. These compound exhibited antimicrobial activity against MRSA at MIC values of 5.1 ± 0.2 and 3.2 ± 0.4 µM [1].

In the continued search for new bioactive compounds, the fungus, *Hypocrea Koningii* PF04 (South China Sea) produced hypocrol A (1) and four known congeners (trichodenol B (2), 4-hydroxyphenethyl acetate (3), 4-hydroxyphenethyl tetradecanoate (4), and 1-oleyltyrosol (5)) [100]. The initial compounds (1–12) had some inhibitory activity but not against MRSA ATCC 43300, while the later compounds showed weak activity against MRSA and other test pathogens. 

A marine sponge (sea fan, *Rumphella* spp.)-associated fungus, *Neosartorya siamensis* KUFA 0017 collected from the coral reef (10 m depth) (Southern Thailand) produced about ten indole alkaloids but only neofiscalin A exhibited inhibitory activity against MRSA and VR *E. faecalis* (MIC, 8 μg/mL) and also prevented their formation of biofilm (BIC = 96 and 80 μg/mL) [5].

A polyketides-producing fungus, *Engyodontium album* strain LF069, associated with a marine sponge (*Cacospinga scalaris*) (Croatia) synthesized compounds named engyodontochones, including another two known compounds. The compounds (1–4) had anti-MRSA activity against clinical isolate of MRSA (DSM 18827), which was even ten times stronger than the reference antibiotic chloramphenicol (Table 2) [101].

From two sponge (*Mycale* sp.)-associated fungi, *Talaromyces tratensis* and *Sporidesmium circinophorum* (Gulf of Thailand), wortmin (1), meso-1,4-bis(4-methoxybenzyl)- 2,3-butanediol (2), and isocoumarin derivative tratenopyrone (3) were synthesized by the former, while the latter produced diphenyl ether derivative, circinophoric acid (4), and other known compounds, catenarin, physcion, mono-methylsoluchrin, and β-ergosterol-5,8-endoperoxide. None of the compounds inhibited *C. albicans* ATCC 10232 (> 512 μg/mL) [102]. 

Anti-MDR sorbicillinoid compounds, saturnispols A–H (1–8) were derived from a sponge (*Dictyonella incisa*)-associated endophytic *T. saturnisporum* DI-IA (Turkey). Strains of *E. faecalis* A4 (VRE) were inhibited at MIC of 1.63 and 12.9 μg/mL, respectively, by only saturnispol F (6) and saturnispol H (8). These may be promising antibiotics for the treatment of Gram-negative bacterial infections [103]. An alkaloid-producing marine sponge (Epipolasis sp)-associated *A. candidus* KUFA0062 (Thailand) synthesized a previously unreported bis-indolyl benzenoid, candidusin D (2e), and a new hydroxypyrrolidine alkaloid, preussin C (5b), including another known fourteen compounds. Only the compound, hydroxypyrrolidine alkaloid preussin (5a), exhibited potent inhibitory activity against MRSA 66/1, and VR *E. faecalis* B3/101 at a MIC value of 32 μg/mL [104].

KumLa et al. identified new chromone derivatives, 1-hydroxy-12-methoxycitromycin (1c) and other constituents previously undescribed like chromone derivates, pyanochromone (3b), spirofuranochromone (4), 7-hydroxy-6-methoxy-4-oxo-3-[(1E)-3-oxobut-1-en-1-yl]-4H-chromene-5- carboxylic acid (5), and a pyranochromone dimer (6), including thirteen known compounds, from the culture broth extract of one of the most studied fungi, *Penicillium erubescens* KUFA0220. In addition to an antibacterial activity of compound 9 against MRSA, compound 8, GKK1032B (8) exhibited inhibitory activity against VR *E. faecalis* B3/101, VR *E. faecium* 1/6/63, and MRSA 66/1 at MIC values of 8, 32, and >64 mg/mL [105].

**Figure 4:**
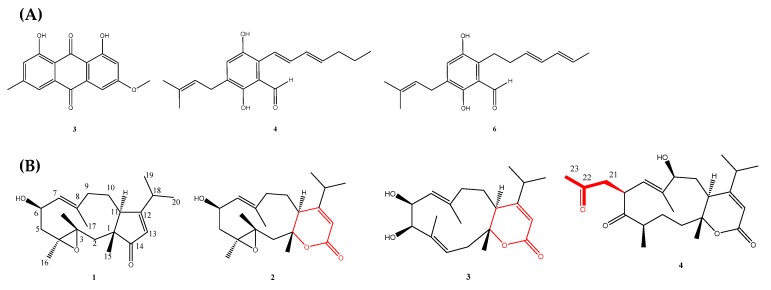
Structures of (**A**) physcion (3), dihydroauroglaucin (4), and isodihydroauroglaucin (6) [106]; and (**B**) dolabellane-type diterpenoids (1−3) and three new atranones (4) [110].

Atlantic sponge (*Grantia compressa*)-associated fungus, *Eurotium chevalieri* MUT 2316 (West Coast of Ireland), produced 10 metabolites with promising antibacterial (Table 3) (Figure 4A) as well as antiviral activities [106]. This study demonstrated and reaffirmed that “One strain, many compounds” (OSMAC) is a powerful method to stimulate and enhance the production of an incredible variety of new secondary metabolites.

The rice culture of *A. niger* from marine sponge (*Haliclona* sp) tissues produced new 4-hydroxy-α-pyrones (nipyrones A–C (1–3)) and a known analogue, germicidin C (4). These compounds, which differ in their functional group substitution and side chain length, exhibited weak to moderate antimicrobial activities with MRSA [107].

An anti-MRSA novel compounds, 4-methyl-candidusin A (1), aspetritone A (2), and aspetritone B (3), including another 15 already known compounds (4–18), were identified in the fermentation culture broth of coral (Galaxea fascicularis)-derived fungus, *A. tritici* SP2-8-1 (Malaysia). Antimicrobial activity testing showed that two strains of MRSA ATCC 43300 and CGMCC 1.12409 were inhibited by aspetritone A (2) and prenylcandidusin at low MIC, while the rest of the compound showed moderate activity [108].

The Coast of Xisha Island’s coral-associated *A. terreus* (China) produced prenylated tryptophan derivative, butenolide derivative, linear aliphatic alcohol, and another nine known compounds (4–12). The prenylated tryptophan derivative, luteoride E (1), has an unusual (E)-oxime group that is rarely found in natural products. Apart from other reported biological activities, only luteoride E (1) showed moderate inhibitory activity against drug resistant pathogens [109].

An antimicrobial dolabellanes and atranones produced by the toxigenic fungus, *Stachybotrys chartarum* TJ403-SS6, synthesized three new dolabellane-type diterpenoids and three new atranones. Compound 2 is structurally related to Compound 3 due to the presence of a 1, 14-seco dolabellane-type diterpenoid skeleton. Additionally, compound 4 is the first C_23_ atranone having a propan-2-one motif linked to a dolabellane-type diterpenoid by a carbon−carbon bond; and compound 5 represents the first example of a C_24_ atranone with a 2-methyltetrahydrofuran-3-carboxylate motif fused to a dolabellane-type diterpenoid at C-5−C-6. Compound 4 (Figure 4B) showed inhibitory activity against only MRSA ATCC 43300 and *C. albicans* ATCC 10231 (Table 3), while other pathogens were not inhibited by all the compounds [110].

A wild crab (*Pachygrapsus crassipes*) associated *Penicillium* sp. ZZ380 produced penicipyrroether A in a PDB medium and pyrrospirone J in a BMPM medium, and other known compounds (3–6, 9 and 10) (Figure S2). Aside from other biological activities, the penicipyrrodiether A, which is a cyclo-condensation product of GKK1032 analogue via the addition of a five-membered ether ring, showed inhibitory activity at MIC value of 5.0 μg/mL against MRSA ATCC 43300, and had anti-glioma proliferation activity [111] while pyrrospirone J inhibited MRSA at a MIC value of 1.7 μg/mL [112]. 

The co-culture of a staghorn Gorgonian fungus, *Rhinocladiella similis* 35, from Luhuitou fringing reefs and actinomycete, *S. rochei* MB037 (South China Sea) resulted in isolation and identification of new fatty acids with rare nitrile group, a new chromone derivative,) and another two known 18-membered macrolides (Table 2). Only compound 1 exhibited anti-MRSA activity against MRSA at MIC value of 0.195 μg/mL [113].

### 3.3. Marine-Derived Antimicrobial Compounds from Algae

A tropical marine *Cyanobacterium*, *Okeania hirsuta* (Republic of Panama), has synthesized a potent polyhydroxy macrolide, bastimolide A. This 40-membered ring macrolide has one 1, 3-diol, one 1, 3, 5-triol, six 1, 5-diols, and one tert-butyl group. The pure form of this compound showed moderate cytotoxic effect and potent antimalarial activity against MDR strains of *P falciparum* (IC_50_ 80–270 nM). These make it a potentially promising lead for antimalarial drug discovery and further research [114].

A linear diterpene-producing brown alga, *Bifurcaria bifurcate* (Ireland) produced a compound with two stereogenic centres, named bifurcatriol (1), which displayed cytotoxicity, and antiprotozoal activity in an *in vitro* study towards a small panel of DR parasites at low dose [115]. Chloroquine-resistant malaria parasite, *P. falciparum* 3D7 strain was very susceptible to a new mono-hydroxy acetylated sterol derivative, 12β-hydroxy-3β, 15α, 16β-triacetoxy-cholest-5-en-7-one (halymeniaol) (1) (IC_50_ = 3.0 μM) produced by an Indian marine red alga, *Halymenia Floresii*. This is the first sterol derivative (aside cholesterol (2)) isolated from a red alga that is non-cytotoxic and has antimalarial potency [116].

Of the four HPLC fractions of an Indian cyanobacteria, *Oscillatoria acuminata* NTAPC05, only fraction 2 showed non-cytotoxic effect and exhibited anti-ESBL production even more than the fourth-generation cephalosporin (MIC > 125 μg/mL1) against well-characterized urinary tract infection-causing ESBL-producing bacteria, *E. coli* U655, *Enterobacter asburiae* B938 and *Stenotrophomonas maltophilia* B929 (MIC/MBC 100 μg/mL). The spectral analysis revealed the presence of monogalactosyldiacylglycerol containing a palmitoyl (3-(3, 4, 5-trihydroxy-6-(hydroxymethyl) tetrahydro-2H-pyran-2-yloxy) propane-1, 2-diyl dipalmitate). This study reported the interactions of two successive H-bonding with Leu198 of TEM1 β-lactamase, according to *in silico* analysis of MGDG-palmitoyl [117].

Baltic Sea brown alga, *Fucus vesiculosus*, was sampled throughout the year to check its potential relation to the bioactivity profile. About 44 compounds were putatively identified, including phlorotannins phlorotannins, phosphatidylcholine, betaine lipids and their lyso derivatives, chlorophylls, and carotenoids. The extract exhibited other biological activities and had no antimicrobial activity against some fungi and ESKAPE panel of human bacterial pathogens except MRSA (100 μg/mL) [118]. In addition to the antiproliferative and neuroprotective activities of kappa-carrageenan extracted from a marine alga, *Hypnea musciformis* (Brazil), it exhibited antimicrobial activity including against MDR *C. albicans* 10231 at IC_50_ value of 147.3 μg/mL [2]. 

An unusual diterpene glycosides (with a sterically encumbered cyclopropane core), peyssonnosides A−B, were produced by *Peyssonnelia* sp. sampled at Solomon Islands, Georgia, USA. The compound showed no cytotoxic effect and exhibited antimicrobial activity against test pathogens, including MRSA at MIC_90_ of 16.7 ± 0.3 and >50 μg/mL, respectively [119].

The methanol extract of microalgae, dinoflagellates, *Amphidinium carterae* led to the isolation of a bioactive polyketides, new amphidinol (amphidinol 22) and two other known amphidinols, with cytotoxic and antifungal properties. It moderately inhibited *C. albicans* ATCC 64124 less than amphidinol A (19 µg/mL) and had no activity against other drug-resistant pathogens, such as *K. pneumoniae* ATCC700603, and MRSA MB5393 [120].

### 3.4. Marine-Derived Antimicrobial Compounds from Invertebrates

Two marine sponges, *Dysidea granulosa* and *Dysidea* spp. (United States), produced potent anti-MRSA polybrominated diphenyl ethers, such as 2-(20, 40-dibromophenoxy)-3, 4, 5-tribromophenol (2) (the most potent), and the compounds exhibited potent and broad-spectrum activity against both Gram-negative and Gram-positive bacteria including, MRSA at MIC value of 0.1 mg/mL. This suggests that these compounds could be useful in drug development in the future [121].

The fraction of the extract of Saudi Red Sea marine sponge, *Hemimycale Arabica*, led to the isolation and identification of new bioactive hydantoin alkaloids, hemimycalins A and B (2 and 3), together with another known compound (Z)-5-(4-hydroxybenzylidene)imidazolidine-2, 4-dione (1). The Compound 2 and 3 are the first natural N-alkylated hydantoins from marine sponge *Hemimycale arabica*. These bioactive metabolites had not only moderate antiproliferative activity, but showed inhibitory activity against MDR *C. albicans* ATCC 14053 (IZD 14–22 mm) [122]. 

The bioactivity-guided fractionation of sponges (*Lamellodysidea* sp. and *Dysidea granulosa* (2)) from Papua New Guinea led the researchers to identify fourteen polybrominated, diphenyl ethers, including a new methoxy-containing compound (8). These compounds displayed strong to moderate antimicrobial activity against test pathogens (Table 2) [123].

The reports on the antibacterial macromolecules from tunicates are relatively few, which led Wang et al. to isolate and identify butenolide metabolites from *Pseudodistoma antinboja* (South Sea, Korea). These class of cadiolide, cadiolides J-M, 1, 3–5, and cadiolide H (2) exhibited considerable antimicrobial activity comparable to the commercial drugs (such as vancomycin and linezolid) against pathogens including four strains of MRSA (1–8 μg/mL) [124].

Wang et al. actioned the isolation and identification of a new N- hydroxy-1, 2, 3, 4-tetrahydro-β-carboline, together with the first example of an acetylenic 1-amino-2-alcohol lipid, distaminolyne A (1) from ascidian, *Pseudodistoma opacum*. The compounds showed moderate activity against pathogens and compound 2 displayed antimalarial activity against a chloroquine-resistant strain (FcB1) of *P. falciparum* (IC_50_ 3.82 ± 0.2 μM) [125]. Another chloroquine-resistant Dd2 strain of *P. falciparum* was very susceptible to known antimalarial sesquiterpenoids (Table 2) produced by marine sponge, *Hyrtios erectus* [4].

Compounds with potent antioxidant and antibacterial activities were purified from an edible portion of Chinese Sea *Arca inflat.* The authors identified the compound to be a new sarcoplasmic calcium-binding protein-like metabolite, named protein (J2-C4), which had moderate inhibitory activity against MRSA (MIC = 750 μg/mL) [126]. This indicated that the protein could be harnessed and developed as a potential food additive.

Okinawan marine sponge (*Pseudoceratina* sp.) produced new bromotyrosine alkaloids, ceratinadins E and F (1, 2) and an already known psammaplysin F (3). The two new compounds have an 8, 10-dibromo-9-methoxy-1, 6-dioxa-2-azaspiro[4,6]undeca-2, 7, 9-trien-4-ol unit with two or three 11-N-methylmoloka’iamine units connected by carbonyl groups, respectively. Compound 1 and 3 exhibited inhibitory activity against drug-resistant *P. falciparum* strains, K1 and FCR3, at an IC_50_ value range of 0.77–3.77 μg/mL while compound F (2) did not display notable antimalarial activity [127].

## 4. Conclusions

About 119 articles were included in this review, and the marine natural products were obtained from 55 bacteria, 48 fungi, 8 algae, and 8 invertebrates. The structure and anti-drug resistant activities of some of the MNPs are summarized in Table 2 and Table 3, and Figure 2, Figure 3 and Figure 4. It is evident from these articles that marine natural products are diverse, abundant, and could evidently serve as a ray of light in the therapy of drug-resistant bacterial, fungal, and parasitic infections, and could also be translated to novel biomedicines. As more MNPs continue to enter clinical trials, more novel compounds with different chemical structures and biological activities are being discovered. However, one of the hurdles in natural product discovery is the high rate of repeated isolation of known compounds. 

It is clear that the majority of isolated MNPs came from bacteria (specifically actinomycetes) and fungi. Most of the compounds were active against drug-resistant pathogens and have other biological properties not discussed here. Genetic engineering of isolated marine microbes through genomic analyses and applying metabolic approach and employing combined biomedical and biotechnological efforts, more novel compounds will be discovered, and the yield of bioactive metabolites will increase. Finally, to make the discovery of and accessibility to natural products easier, there is a need to develop automated and more affordable techniques for the isolation and identification of marine natural products.

## Figures and Tables

**Table 1:** Antibiotic-resistant “priority pathogens”.

Microorganisms	Drug	Categories and Priority
*Acinetobacter baumannii*	carbapenem-resistant	**1st and critical**
*Pseudomonas aeruginosa*	carbapenem-resistant	
*Enterobacteriaceae*	carbapenem-resistant, extended spectrum beta-lactamases (ESBL)-producing	
*Enterococcus faecium*	vancomycin-resistant	**2nd and high**
*Staphylococcus aureus*	methicillin-resistant, vancomycin-intermediate and resistant	
*Helicobacter pylori*	clarithromycin-resistant	
*Campylobacter* spp.	fluoroquinolone-resistant	
*Salmonellae*	fluoroquinolone-resistant	
*Neisseria gonorrhoeae*	cephalosporin-resistant, fluoroquinolone-resistant	
*Streptococcus pneumoniae*	penicillin-non-susceptible	**3rd and medium**
*Haemophilus influenzae*	ampicillin-resistant	
*Shigella* spp.	fluoroquinolone-resistant	

Source: https://www.who.int/news-room/detail/27-02-2017-who-publishes-list-of-bacteria-for-which-new-antibiotics-are-urgently-needed, https://www.who.int/news-room/fact-sheets/detail/antimicrobial-resistance.

**Table 2:** Some of the marine natural products active against pathogens at low concentration.

Drug Resistant Microbe	Compound (Activity)	Class	Source	References
MRSA SK1	AMA11, AMA12 and AMA21 (0.5–4 μg/mL), AMA11 CE 6 (quinoxaline-2-carboxamide) (32 μg/mL), AMA11 CE 7 (3-nitro-1,2-benzenedicarboxylic acid and quinoxaline-2-carboxamide) (0.25 μg/mL)	Quinone	Mangrove sediment-derive *Streptomyces* spp.	[27]
MRSA	3-amino-27-demethoxy-27-hydroxyrifamycin S (1), 3-amino-rifamycin S (2), sporalactam A (3), and sporalactam B (4) (0.0009, 0.0008, 7.0, and 1.8 μM)	Macrolides	Sediment *Micromonospora* sp. RJA4480	[28]
MRSE, MRSA, VR *E. faecium* ATCC 700221 and VR *E. faecalis* ATCC 51299	Quinomycin G (1) and dipeptide, cyclo-(L-Pro-4-OH-L-Leu) (2) (16–32 μg/mL) and Echinomycin (0.25–0.5 μg/mL)	Cyclic dipeptide	Sponge (*Gelliodes carnosa*) *Streptomyces* sp. LS298	[57]
MRSA MB5393	napyradiomycin, MDN-0170 (1), 4-dehydro-4a-dechloronapyradiomycin A1 (2), napyradiomycin A1 (3) and 3-chloro-6,8-dihydroxy-8- -lapachone (4) (> 64, 4–8, 0.5–1, and >64 μg/mL)	Napyradiomycin	*Streptomyces* sp. strain CA-271078 from sea shore ascidian	[58]
MRSA ATCC 43300	Actinomycins D (1), V (2), and X0 (3) (0.08, 0.08, and 0.61 μM)	Peptide	*Streptomyces* sp. ZZ338 from squirts	[61]
MRSA	penicillstressol (7), isopenicillstressol (8), and 0Z-isocitreoviridinol (5) (0.5–1 μg/mL)	Polyketide	*Penicillium* sp. BB1122 from marine sediment	[74]
MRSA and MRSE	Acremolin C (1), cyclo-(L-Trp-L-Phe) (2), 4-hydroxyphenyl acetic acid (3), (7S)-(+)-hydroxysydonic acid (4) and (7S, 11S)-(+)-12-hydroxysydonic acid (5) (32, 1 > 128, 1, and 1 μg/mL) and (16, 0.5, >128, 0.5, and 0.5 μg/mL)	Alkaloid	*Aspergillus sydowii* SP-1 from antarctic marine sediment (50 cm)	[77]
MRSA DSM 18827	Engyodontochone A (2), B (4), C (5), D (6), E (7), and F (8) (0.17–6.74 ± 0.02–0.30 μM)	Polyketides	Sponge-derived *Engyodontium album* Strain LF069	[101]
MRSA	borrelidins J (0.195 μg/mL) and K (1 and 2), and others 7-methoxy-2,3-dimethylchromone-4-one (3), borrelidin (4) and borrelidin F (5).	Fatty acids, macrolides	*S. rochei* MB037 and *Rhinocladiella similis* 35 derived from sponge (Dysidea arenaria) and staghorn gorgonian	[113]
MRSA ATCC 43300 and VR *E. faecium* ATCC 51299	14 polybrominated, diphenyl ethers (methoxy-containing compound (8)) (0.078–> 50 μg/mL)		Dictyoceratidsponges *Lamellodysidea* sp. and *Dysideagranulosa* (2)	[123]
CR- Dd2 strain of *P. falciparum*	smenotronic acid (1), ilimaquinone (2), and pelorol (3) (3.51, 2.11 and 0.8 μM)	Sesquiterpene quinone	marine sponge, *Hyrtios erectus*	[4]

NA—not active; MDR—multi-drug resistant—MRSA: methicillin-resistant *Staphylococcus aureus;* MRSE—methicillin-resistant *Staphylococcus epidermidisů* VR—vancomycin–resistant; ESBL—extended spectrum beta-lactamases, CR—chloroquine-resistant.

**Table 3:** Some of the most recent (2019) marine natural products (MNPs) with broad antimicrobial activity or active against multidrug-resistant pathogens.

Drug Resistant Microbe	Compound (Activity)	Class	Source	References
MDR *E. faecalis* CCARM 5172, *E. faecium* CCARM 5203, *E. coli* CCARM 1009, *S. typhimurium* CCARM 8250, *S. aureus* CCARM 3090	(1, grincamycin L) 3.12–≥50 μg/mL and angucycline derivatives (2 (3.12–≥50 μg/mL) and 3) (Figure 2A)	Polyketides	Deep sea-sediment derived *S. lusitanus*	[42]
*S. aureus* WC 25 V 880854, *E. coli* (ESBL 4345), ESBL *K. pneumoniae* ATCC70063, *A. baumannii* MDR 4414 and *E. faecium* VRETC 773	3-methylpyridazine, n-hexadecanoic acid, indazol-4-one, octadecanoic acid and 3a-methyl-6-((4-ethylphenyl) sul (6.25–100 μg/mL) (Figure 2B)	Alkaloids	*Streptomyces* sp. Al-Dhabi-90 from marine samples	[43]
MRSA ATCC 43300, VR *E. faecalis* ATCC 51213	Salinaphthoquinones B and D (2 and 4) (16–125 μg/mL); A, C, and E (1, 3, and 5) (>125 μg/mL) (Figure 2C)	Quinone	*Salinispora arenicola* from marine sediments	[44]
*E. coli* ATCC 35218, MRSA ATCC 33591, *E. faecalis* ATCC 310682; and *K. pneumonia* ATCC 700603, *P. aeruginosa* ATCC 2774, *A. baumannii* ATCC 19606	Quinomycin A (1.10–3.60 cm and NA) and monosulfoxide quinomucin (Figure 2D)	Cyclic octapeptide	*Streptomyces* sp. B475 from mangrove soil	[45]
MRSA ATCC NR-46171 and MRSA ATCC-46071	4-bromophenol (15.62 and 7.81 μg/mL) and Bis (2-ethylhexyl) phthalate (125 and 15.62 μg/mL) (Figure 2E)	Bromophenol derivative and phthalate ester	*Nocardiopsis* sp. strain SCA21 from marine sediment	[47]
*M. tuberculosis* MDR-1 ATCC 14595 and MDR-2 14615, MDR *C. urealyticum* 1492, VR *E. faecalis* ATCC (NJ3) 51299 and MRSA ATCC 43300	Desertomycin G (16, 16, <0.25, 8, and 4 μg/mL) (Figure 3A)	Macrocycles	*S. althioticus* MSM3 from marine Intertidal macroalgae (*Ulva* sp.)	[55]
*C. albicans* 10231	(2S,2”S)-6-lavandulyl-7,40-dimethoxy-5,20-dihydroxyl flavanone (1), (2S,2”S)-6-lavandulyl-5,7,20,40-tetra hydroxylflavanone (2), and (2”S)-50-lavandulyl-20-methoxy-2,4,40,60-tetrahydroxylchalcone (3) (1–32 μg/mL) and compounds 4–10 (>256 μg/mL)	Lavandulylated flavonoids	*Streptomyces* sp. G248 from marine Sponge (*Halichondria panicea*)	[70]
MDR *- H. pylori*	TM1: (i) 1, 3-dione-5, 5-dimethyl- cyclohexane (17.18 ± 1.25 μg/mL), (ii) 2-enone-3hydroxy -5,5-dimethylcylo-hex and TM2 4H-1,3-dioxin-4-one-2,3,6-trimethyl (14.67 ± 0.15 μg/mL) (Figure 3C)		*Trichoderma atroviride* strain KNUP001 from coastal wetland sediment	[7]
MRSA ATCC 43300 and ATCC 33591, VR *E. faecalis* ATCC 51299	peninaphones A–C (1–3) (12.5–≥50 μg/mL) and known bis-naphtho-γ-pyrones (compounds 4 and 5) (≥50 μg/mL) (Figure 3F)	Naphtho-γ-Pyrones polyketide	*Penicillium* sp. HK1-22 from mangrove rhizosphere soil	[97]
MRSA Monza-PFI, fluoroquinolone-resistant *S. aureus* Monza-FD1, and a macrolide-resistant *S. pneumoniae* Monza-82	Echinulin (1), Neoechinulin A (2) (>128 μg/mL), Physcion (3) (16–>32 μg/mL), Dihydroauroglaucin (4) (8–>128 μg/mL), Flavoglaucin (5) (>32 μg/mL), Isodihydroauroglaucin (6) (4–64 μg/mL) (Figure 4A), Neoechinulin D (7), Asperflavin (8), Cinnalutein (9) and Cyclo-L-Trp-L-Ala (10) (32–≥128 μg/mL)	Peptides	*Eurotium chevalieri* MUT 2316 from marine Sponge (*Grantia compressa*)	[106]
MRSA ATCC 43300, *C. albicans* ATCC 10231	Atranones D (4) (8 and 32 μg/mL) (Figure 4B)	Dolabellanes and Atranones	Marine-derived fungus, *Stachybotrys chartarum* TJ403-SS6 from coral (*Sarcophyton subviride*)	[110]
ESBL *E. coli* ATCC 35218, ESBL K. pneumoniae ATCC 700603,	dolabellane-type diterpenoids (1–3) and atranones (5, 6) (≥100 μg/mL)			

NA—not active; MDR—multi-drug resistant; MRSA—methicillin-resistant *Staphylococcus aureus;* MRSE—methicillin-resistant *Staphylococcus epidermidisů* VR—vancomycin–resistant; ESBL—extended spectrum beta-lactamases.

## Supplementary Materials

The following are available online at https://www.mdpi.com/1660-3397/18/3/145/s1, Figures S1 and S2: the structures of some of compounds reported in 2019 and Table S1: summary of some of the articles on marine natural products tested against drug-resistant pathogens.
