# Supplementary material for: Antibiotics Development and the Potentials of Marine-Derived Compounds to Stem the Tide of Multidrug-Resistant Pathogenic Bacteria, Fungi, and Protozoa
Source: Mar Drugs. 2020 Feb 28;18(3):145. doi: 10.3390/md18030145 (PMC7142797; doi:10.3390/md18030145)
Supplement: Supplementary file 1 [file marinedrugs-18-00145-s001.pdf]

Supplementary Materials: Tables

Table 1: Some of isolated compounds with good therapeutic importance

Drug resistant Microbe	Activity	Compound	Class	Source	References
C. albicans ATCC 10231	0.5 -5 mg/mL	4-Dichloromethyl-5 6-epoxy-2-methoxy-4-methyl-2-cyclohexenone, derivative of 1,2,4-triazol [1,5-a] pyrimidine,5,7-dimethyl-2-phenyl-, vinylbital, bruceantin, digitoxigenin 2-isopentyl and isoquinoline-1-carbonitrile, and 2, 2-propyl-N-ethylpiperidine; 1,3-cyclopentanedione		Mangrove *Streptomyces* sp. SMS_SU21	[19]
C. albicans ATCC 10231	8 and 64 µg/mL	(3S, 8aS)-3-(4-hydroxybenzyl) hexahydropyrrolo[1,2-a] pyrazine-1,4-dione [Cyclo(L-Pro-L-Tyr)], and 3-benzyl hexahydropyrrolo [1,2-a] pyrazine-1,4-dione [Cyclo(L-Pro-L-Phe)]	Peptides	Mangrove soil sendiments, *Pseudonocardia endophytica* VUK-10	[24]
MRSA	16, 16, 16 - 32, 32, and 32 µg/mL	stremycin A (1) and B (2), 2-[2-(3,5-dimethyl-2-oxo-cyclohexyl)-6-oxo-tetrahydro-pyran-4yl]-acetamide (3), cyclo[L-(4-hydroxyprolinyl) -L-leucine] (4), 2-methyl-3H-quinazoline-4-one (5), and menthane derivative, 3-hydroxymethyl)-6-isopropyl-10,12-dioxatri -cyclo[7.2.1.0]dodec-4-en-8-one (6)	Angucyclines polyketides	marine sediment *S. pratensis* NA-ZhouS1	[30]
MRSA ATCC 43300 and CGMCC 1.12409	32.4 0.1 - 15.2 0.4 µg/mL	chaephilone C (1), chaetoviridides A - D (1 - 4), chaetoviridin A (5), chaetomugilin D (7) and cochliodone A (8).	Chlorinated azaphilone polyketide	*Chaetomium* sp. NA-S01-R from Deep sea (4050 m)	[76]
	>50 µg/mL	chaetoviridine E (6)			
MDR *S. aureus* 6538P	1.0 - 0.5 mg/mL	MUT 4861 (Scopularide A (8)) and MUT 4865 (1,3-dihydroxy-2-amino-6,17-docosadiene structure)	Cyclodepsipeptide and tetracyclic diterpene	Fungi, *Microascacea* sp. MUT 4861 and *Beauveria bassiana* MUT 4865 from marine green alga, *Flabellia petiolata*	[6]
MDR *B. metallica* LMG 24068	0.5 - 0.25 mg/mL				
MDR *P. aeruginosa* PA01	0.5 - 2.0, and >2.0 mg/mL				
MDR *K. pneumoniae* DF12SA	>2.0 - 2.0 - 1.0 and 1.0 - 0.5 - >2.0 mg/mL				

Supplementary Materials: Figures

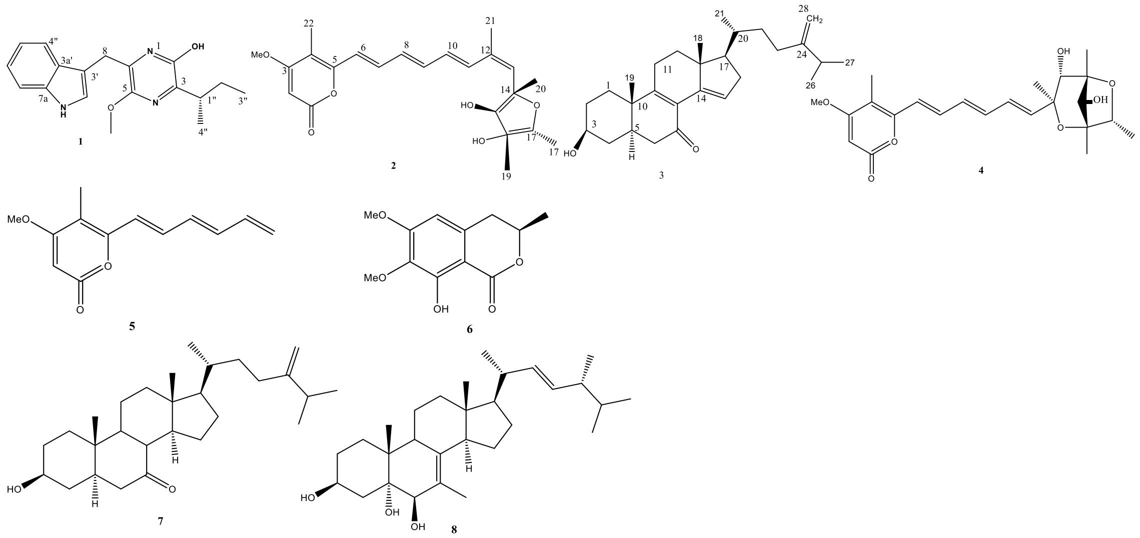

Figure S1: new pyrazine derivative, trypilepyrazinol (1), a new -pyrone polyketide, (+)-neocitreoviridin (2), and a new ergostane analogue, 3-hydroxyergosta-8,14,24(28)-trien-7-one (3), and other five known compounds [82].

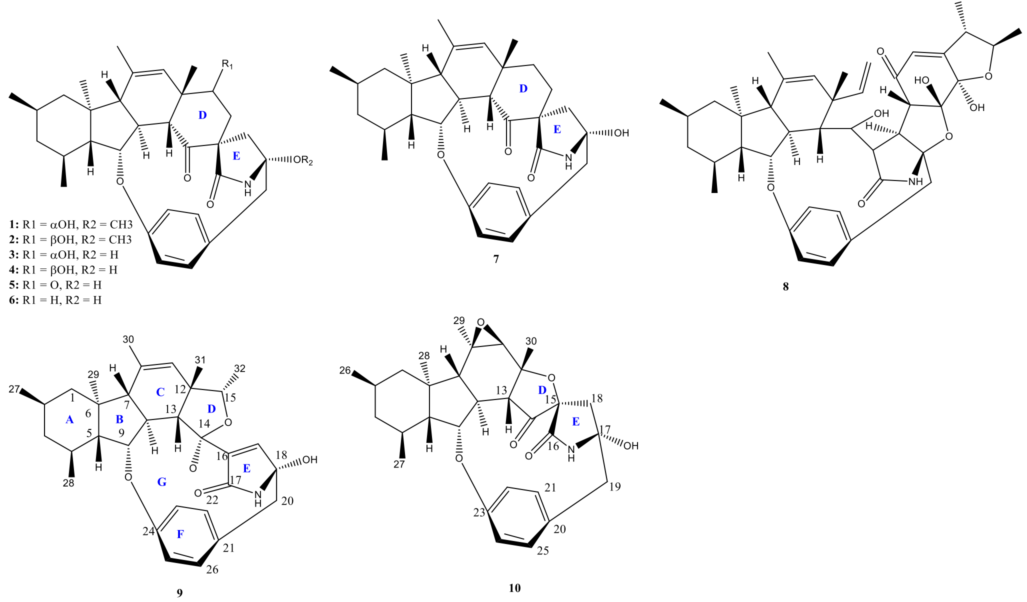

Figure S2: Structures of 10 out of 18 compounds isolated from the cultures of *Penicillium* sp. ZZ380. penicipyrroether A (9) and pyrrospirone J (10) [112].
